# Anemia and Iron Deficiency in Cancer Patients: Role of Iron Replacement Therapy

**DOI:** 10.3390/ph11040094

**Published:** 2018-09-30

**Authors:** Fabiana Busti, Giacomo Marchi, Sara Ugolini, Annalisa Castagna, Domenico Girelli

**Affiliations:** Department of Medicine, Section of Internal Medicine, University of Verona, and EuroBloodNet Referral Center for Iron Disorders, Azienda Ospedaliera Universitaria Integrata Verona, Policlinico G.B. Rossi, 37134 Verona, Italy; fabiana.busti@univr.it (F.B.); markallbutone@gmail.com (G.M.); sara.ugolini@univr.it (S.U.); annalisa.castagna@univr.it (A.C.)

**Keywords:** iron deficiency, anemia, cancer, hepcidin, patient blood management

## Abstract

Anemia in cancer patients is quite common, with remarkable negative impacts on quality of life and overall prognosis. The pathogenesis is complex and typically multifactorial, with iron deficiency (ID) often being a major and potentially treatable contributor. In turn, ID in cancer patients can be due to multiple concurring mechanisms, including bleeding (e.g., in gastrointestinal cancers or after surgery), malnutrition, medications, and hepcidin-driven iron sequestration into macrophages with subsequent iron-restricted erythropoiesis. Indeed, either absolute or functional iron deficiency (AID or FID) can occur. While for absolute ID there is a general consensus regarding the laboratory definition (that is ferritin levels <100 ng/mL ± transferrin saturation (TSAT) <20%), a shared definition of functional ID is still lacking. Current therapeutic options in cancer anemia include iron replacement, erythropoietic stimulating agents (ESAs), and blood transfusions. The latter should be kept to a minimum, because of concerns regarding risks, costs, and limited resources. Iron therapy has proved to be a valid approach to enhance efficacy of ESAs and to reduce transfusion need. Available guidelines focus mainly on patients with chemotherapy-associated anemia, and generally suggest intravenous (IV) iron when AID or FID is present. However, in the case of FID, the upper limit of ferritin in association with TSAT <20% at which iron should be prescribed is a matter of controversy, ranging up to 800 ng/mL. An increasingly recognized indication to IV iron in cancer patients is represented by preoperative anemia in elective oncologic surgery. In this setting, the primary goal of treatment is to decrease the need of blood transfusions in the perioperative period, rather than improving anemia-related symptoms as in chemotherapy-associated anemia. Protocols are mainly based on experiences of Patient Blood Management (PBM) in non-oncologic surgery, but no specific guidelines are available for oncologic surgery. Here we discuss some possible approaches to the management of ID in cancer patients in different clinical settings, based on current guidelines and recommendations, emphasizing the need for further research in the field.

## 1. Anemia in Cancer: Prevalence, Pathophysiology and Prognostic Impact 

Anemia is a common and potentially detrimental complication in cancer patients, that compromises quality and expectancy of life. Prompt recognition and management has been associated with improvement of clinical outcomes, favoring also a better tolerance and response to antitumoral therapy.

In a prospective epidemiological survey conducted in 34 European countries (European Cancer Anemia Survey (ECAS)) involving about 15,000 subjects diagnosed with solid or hematological tumors between 2001 and 2002, anemia (Hb <12 g/dL) was present in approximately 39% of patients at enrolment, and the overall prevalence increased to 67% during the 6 months’ follow-up [1]. In most cases anemia was mild (defined as Hb >10 g/dL according to the National Comprehensive Cancer Network guidelines [2]), but Hb values lower than 10 g/dL were detected in 10% of patients at baseline and in 39.9% of patients during the follow-up. In another literature review published in 2004, the prevalence of cancer anemia was highly variable (from 30% to about 90%), although this was partially due to different cut-offs used for the diagnosis (Hb <9 *versus* <11 g/dL) [3]. Anemia occurs more frequently in patients with tumor recurrence, at an advanced stage of disease (i.e., from 40% of patients with early-stage colon tumors to nearly 80% of patients with advanced disease), and in those receiving antitumoral treatment. Furthermore, its prevalence varies according to the type of cancer and is higher in patients with hematologic malignancies, such as multiple myeloma and lymphoma. Among solid tumors, the highest incidence of anemia has been reported among lung and breast tumors, followed by gynecological and gastrointestinal malignancies [4].

**Figure 1:**
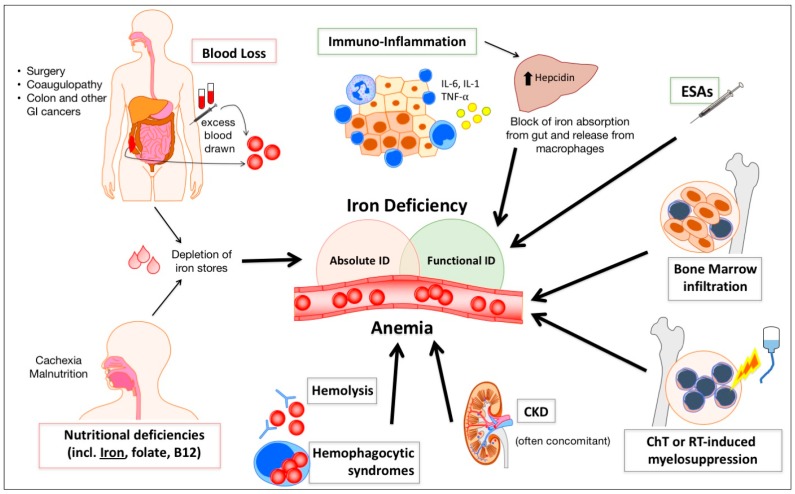
Schematic illustration of the main mechanisms contributing to anemia and iron deficiency in cancer patients. Blood losses due to tumor growth (especially in gastrointestinal cancers) or after surgery, possibly favored by concomitant coagulopathy, and inadequate iron intake due to cachexia and malnutrition lead to absolute iron deficiency (ID). Inflammation increases hepcidin synthesis in the liver, leading to functional ID. Treatment with erythropoiesis stimulating agents may contribute to functional ID, determining a discrepancy between iron need for erythropoiesis and iron supply from the stores. Other factors, such as bone marrow infiltration by tumor cells, myelosuppression caused by chemo- or radio-therapy, and concomitant chronic kidney disease (CKD), often contribute to the development of anemia in cancer patients.

The pathogenesis of cancer anemia is complex and multi-factorial and, even in the same patient, different mechanisms can prevail at different times (e.g., after surgery or chemotherapy (ChT)) [5]. Anemia can develop as a consequence of malnutrition and malabsorption (leading to iron and other nutritional deficiency, e.g., folates or vitamin B12), acute and/or chronic bleeding, systemic inflammation, metastatic infiltration of bone marrow, and therapy-related myelosuppression. Less frequently, cancer anemia may derive from other mechanisms including hemolysis, hemophagocytosis, and hypersplenism (Figure 1). However, not all causes are of equal importance in different cancers. For example, overt or occult bleeding and iron deficiency are often prominent in gastrointestinal, urogenital and gynecological tumors, while bone marrow replacement by metastases is relatively frequent in breast and prostate cancer [6]. Anemia can also be attributed to a decline in endogenous erythropoietin (EPO) production (e.g., during concurrent chronic kidney disease) or a reduction in bone marrow response to EPO [7].

Cancer anemia may be associated with a broad spectrum of symptoms, depending on its severity and rapidity of development. Fatigue is the most debilitating symptom [8,9], followed by impaired mental capacity, confusion and depression, especially in elderly people. Nausea, loss of appetite, dyspnea, syncope and falls can also occur, particularly in patients with comorbidities such as cardio-pulmonary and renal dysfunctions. Of note, the decrease in quality of life (QoL) is particularly evident when Hb drops between 11.5 and 10 g/dL, which is classically considered as mild anemia and not perceived as a problem by most doctors [10]. Not infrequently, symptoms related to anemia represent the first alarm sign of an occult neoplasm, as it is classically observed in patients with colon cancer.

Anemia has been recognized as an independent predictor of poor prognosis in cancer patients. In a comprehensive systematic review of 60 studies evaluating survival, there was a 65% overall increase in the risk of mortality in cancer patients with anemia compared with those without anemia. This ranged from 19% in subjects with lung neoplasia to near 75% in patients with head and neck carcinoma or lymphoma [11]. The impact of anemia on survival has been related to delay in initiating, or failure to complete, the ChT regimens. A poorer response to anticancer treatments has also been evoked, as cytotoxicity induced by radiotherapy (RT) and some ChT agents require adequate tissue oxygen levels. Moreover, a decrease in the oxygen (O_2_) transport capacity of the blood can facilitate intra-tumoral hypoxia, with activation of Hypoxia Inducible Factors (HIFs). Indeed, HIFs are considered master regulators of cancer progression [12,13,14,15] by up-regulation of target genes involved in angiogenesis, immune evasion, and metabolic reprogramming of cancer cells [16], making them resistant to ChT and RT [17,18].

Given the impact of anemia on QoL, disease progression and survival in cancer patients, adequate treatment strategies appear of paramount importance. Several studies have shown that the treatment of cancer anemia determines a marked improvement in QoL [19], particularly among patients with mild-to-moderate anemia. It may also have the potential to improve anti-cancer treatment tolerability and efficacy, with a possible impact on prognosis [9,20,21].

## 2. Blood Transfusions and Erythropoietic Stimulating Agents: A Double-Edged Sword

Management of anemia in cancer patients often requires a multidisciplinary approach, aimed at recognizing and treating the underlying cause (whenever possible) and at restoring hemoglobin levels. The above-mentioned ECAS survey showed that anemia was treated in less than 40% of patients, mainly with Erythropoietic Stimulating Agents (ESAs) and blood transfusions [1]. Indeed, until recently, blood transfusions have represented the most commonly employed treatment for cancer anemia. Whilst effective in providing an immediate increase in Hb, the benefits of transfusions are transient and concerns about their negative effects have prompted clinicians to consider alternative treatment approaches. Indeed, transfusions are potentially associated with important adverse effects, such as anaphylactic reactions, transfusion-related acute lung injury (TRALI), circulatory overload, iron accumulation, infectious pathogens transmission, as well as an increased susceptibility to infections because of transfusion-related immunosuppression [22]. Blood transfusions have been independently associated to an increased risk for adverse outcomes also in cancer patients undergoing surgery. Numerous studies and meta-analysis have observed that cancer patients receiving transfusions during the perioperative period have an increased risk for mortality, morbidity and tumor recurrence [23,24,25,26]. A systematic review has shown that restrictive transfusion regimens (e.g., Hb thresholds set at 7–8 g/dL) in oncological surgery decrease blood utilization without increasing mortality and morbidity [27]. However, whether or not a restrictive regimen in cancer patients undergoing surgery is as safe as a liberal regimen remains debated, especially in critically ill patients [28,29,30].

On the other hand, since the 1990s, the development of recombinant human erythropoietin has represented an important alternative to blood transfusions for treating anemia. Earlier studies indicated that ESAs reduced transfusion requirements in cancer patients [31,32], as well as relieved the symptoms of anemia and improved QoL [32]. However, concerns on the use of ESAs in cancer patients arose in the late 2000s. Meta-analyses suggested that use of ESAs was associated with an increased risk of venous thromboembolism and mortality [33,34], particularly if target Hb levels exceeded 12 g/dL. An increase in mortality and/or disease progression were reported particularly in studies where ESAs were used off-label, such as in anemic patients receiving RT only [35], or receiving neither RT nor ChT [36,37]. The potential for ESAs to promote tumor progression or recurrence, possibly by stimulation of EPO receptors expressed by tumor cells [38], has long been debated. However, few studies have specifically addressed this issue, and the sparse preclinical and clinical data does not appear to support a direct or indirect effect of ESAs on tumor growth and disease progression [39,40,41]. More recent studies have given reassuring data when use of ESAs is restricted to patients receiving ChT with lower target Hb levels [42,43]. Anyway, at present there is a consensus that ESAs are not indicated in anemic cancer patients who are not receiving ChT (except for low-risk myelodysplastic syndromes) [44], while controversy remains in patients receiving ChT when cure is the goal. 

Given the potential risks related to the use of blood transfusions and ESAs, and the growing knowledge regarding iron pathophysiology and its implication in cancer anemia, IV iron administration represents a promising, potentially valuable, therapeutic approach.

## 3. Iron Deficiency in Cancer Patients: A Common Problem, but Difficult to Define 

### 3.1. Impaired Iron Stores and Utilization in Cancer Patients

ID with or without anemia is a frequent complication in cancer patients: in a single center survey involving >1500 patients with solid and hematological malignancies, the prevalence of ID (defined as TSAT <20%) was approximately 42% [45]. Subjects with pancreatic, colorectal and lung tumors were more frequently affected by ID, as well as patients with an advanced stage of disease or treated with chemotherapeutic agents. ID by itself, even in the absence of anemia, may be associated with impaired physical function, weakness, and fatigue, which can be ameliorated by iron therapy [46]. Cancer patients can have either functional or absolute iron deficiency (FID or AID, respectively). FID is most frequent [47] and represents a condition in which iron stores are apparently adequate, but there is insufficient iron supply for erythropoiesis. FID is mainly due to the release of cancer-associated pro-inflammatory cytokines (e.g., IL-6, IL-1, TNF-α, and interferon-γ), that upregulate hepcidin synthesis in the liver [48,49]. Hepcidin is a small peptide hormone that represents the central regulator of systemic iron homeostasis. It acts by inhibiting the only known iron exporter—ferroportin, and hence reducing iron flows into plasma from macrophages involved in recycling of senescent erythrocytes, duodenal enterocytes involved in iron absorption from the diet, and hepatocytes iron stores [50]. FID is one of the major contributors to the so-called anemia of chronic disease [4,51], including cancer [45]. It may also develop during increased erythropoiesis mediated by ESAs therapy and, not infrequently, it is the cause of ESAs unresponsiveness.

On the other hand, AID is a condition in which iron stores are actually depleted. Nutritional deficiencies (e.g., tumor-induced anorexia or malabsorption in gastric or pancreatic cancer; or after resection of intestinal tumors) and, especially, blood losses (manifest or occult, e.g., in colon cancer or after surgery) contribute to AID in cancer patients.

Chronic kidney disease is a relatively frequent comorbidity in cancer patients, which can contribute to anemia not only through a reduction of EPO synthesis, but also through increased hepcidin levels leading to iron trapping within the macrophages, and eventually to FID [52,53].

Figure 1 summarizes the main mechanisms leading to anemia and perturbation of iron metabolism in cancer patients.

### 3.2. Characterization of Iron Status in Cancer Patients 

In healthy individuals, ferritin reflects the status of iron stores, while various other parameters such as TSAT, percentage of hypochromic erythrocytes (%HYPO), Hb-content of reticulocytes (CHr), and soluble transferrin receptor (sTfR), reflect the amount of biologically available iron. Unfortunately, most of these parameters are altered in cancer patients and the diagnosis of ID in this setting poses multiple challenges. Notably, ferritin is an acute-phase protein and may not correlate with iron stores during inflammatory states and liver disease, conditions that are relatively frequent in cancer patients. Thus, with respect to AID, while in otherwise normal individuals a serum ferritin level of <20–30 ng/mL is virtually diagnostic, in cancer patients a higher ferritin cut-off (e.g., <100 ng/mL) appears more reliable [54], as suggested in other chronic inflammatory conditions such as kidney disease or heart failure [55]. Moreover, some studies noted that a ferritin level of 100 ng/mL might be a good threshold to identify patients better responsive to IV iron therapy [56,57]. 

On the other hand, the ferritin cut-off for defining FID or, in a more practical way, for deciding whether or not iron supplementation should be considered in cancer patients, is much more debated and still unresolved. Indeed, most experts [58] and the few guidelines available [2,54] recommend testing both ferritin and TSAT, and considering FID when TSAT is <20% with variable ferritin levels ranging from 100 up to 800 ng/mL. However, TSAT also has some limitations in cancer patients, since reduced transferrin levels due to inflammation and/or malnutrition can result in falsely normal to high values.

An increased sTfR and a reduced sTfR/log ferritin index have been reported as possible indicators of FID [51], but sTfR lacks standardization [59] and the interpretation of this cumbersome equation in clinical practice is far from ideal [47,50]. Finally, although %HYPO and CHr are cheap and theoretically informative tests (with thresholds of >5% and <28 pg, respectively), only few laboratories provide such data and most physicians overlook them. 

Measurement of circulating hepcidin is a promising tool for assessing iron status [60]. While opposing stimuli can influence hormone levels, with inflammation increasing and ID decreasing them, ID tends to prevail when both are present [61]. Indeed, low hepcidin has been proven effective in distinguishing ID anemia from anemia of chronic disease in patients with different inflammatory disorders, like rheumatoid arthritis [62] and inflammatory bowel diseases [63]. Similar results have been reported in a small study on patients with cancer anemia [64], but further validation is needed. Of note, low hepcidin levels may be useful not only for diagnosis of ID, but also for predicting response to iron treatment [65]. This has been shown also in a study on patients with ChT-associated anemia treated with darbepoetin [66]. Whether or not this may be true also in cancer patients not treated with ESAs remains to be demonstrated. Currently, at international level, many laboratories are putting efforts into harmonizing the heterogeneous hepcidin assays in order to define standardized cut-offs, and hence allowing full implementation in clinical practice [60,67].

## 4. Evidence for Iron Treatment in Cancer Anemia

To date, the majority of clinical trials have investigated the effects of iron treatment in combination with ESAs, demonstrating multiple benefits in term of hematological response (increase in Hb levels) [68,69,70,71,72,73,74], improvement of QoL [68], reduction of transfusions requirements [71] and lowering ESAs doses [69], as compared to treatment with ESAs only. A meta-analysis of eleven trials, including more than 1600 cancer patients randomized to treatment with IV iron, confirmed the activity of iron either as sole treatment or in combination with ESAs [75]. In particular, IV iron significantly increased hematopoietic response and decreased the rate of blood transfusions in trials both with and without ESAs. The increase in the hematological response rates correlated with total IV iron dose, regardless of baseline iron status. Similar results emerged in another meta-analysis, showing an increase of hematological response rates and a decrease of transfusion requirements with the addition of IV iron to ESAs therapy. In contrast, treatment with oral iron was not effective [76]. Growing evidence seems to confirm benefits of IV iron alone, particularly when the newer, third-generation compounds are used (for an extensive review on modern iron replacement therapy see [77]). Initially, three small studies in gynecological cancer patients receiving chemo-radiotherapy showed significant reductions of transfusion needs after IV administration of iron sucrose [78,79,80]. Subsequently, an observational study with ferric carboxymaltose (FCM) (median dose 1000 mg), including more than 600 patients with active malignancies and cancer anemia and/or ChT-induced anemia, revealed a similar hematological response in patients treated with IV iron alone as compared to the combination with ESAs [56]. Hb increase was higher in patients with low initial Hb levels (<10 *versus* ≥10 g/dL) and in those with serum ferritin levels <100 ng/mL. Noteworthy, patients with ferritin up to 500 ng/mL but low transferrin saturation also benefited from FCM treatment, highlighting that cancer patients can effectively respond to IV iron even when FID is present. In this line, a small prospective randomized controlled trial evaluated FCM without ESAs for correction of anemia in lymphoma patients with FID (defined as TSAT ≤20% and ferritin >40 ng/mL in men and >30 ng/mL in women). Patients in the FCM arm had a mean Hb increase significantly higher as compared to controls at week 8 [81]. IV FCM administration as monotherapy also effectively stimulated hematological response in a group of patients with gastrointestinal malignancies [57]. In agreement with Steinmetz et al., this trial suggested baseline ferritin levels <100 ng/mL as the predictor of response. Treatment with FCM has also been associated with a significant increase of QoL in patients with various solid tumors [82].

At variance with IV iron, oral iron has not been associated with consistent clinical or hematological improvement in cancer patients [68,70,75,76]. In cancer patients, concurrent inflammation, gastrointestinal discomfort, polypharmacy, and malabsorption make oral iron a poorly suitable choice. 

## 5. The Emerging Role of Patient Blood Management Programs in Oncologic Surgery and the Contribution of Iron Treatment

In recent years, the increasing awareness on the possible adverse effects related to the use of blood transfusions, especially in the perioperative period, led to the implementation of so-called programs of Patient Blood Management (PBM). This term refers to patient-centered, multi-disciplinary activities that promote safety, appropriateness, and evidence-based use of blood [83], with the aim of reducing the short and long-term adverse consequences related to blood transfusions. The PBM pillars include the optimization of erythropoiesis (e.g., by correcting ID) and hemostasis, as well as the use of anesthetic/surgical technologies aimed at minimizing of blood loss.

Preoperative anemia is the major predictive factor for allogeneic blood transfusion in surgical patients, therefore its optimal management represents one of the cornerstones of PBM programs [84] and, from 2010, is recommended by the World Health Organization (WHO). Multiple studies have demonstrated the effectiveness of preoperative anemia management, mainly through iron supplementation in elective orthopedic [85] and cardiothoracic surgery [86], not only by limiting blood transfusions, but also by reducing postoperative complications (such as acute kidney failure or infection) and length of hospitalization. By contrast, robust evidence for a similar efficacy in oncologic surgery is still lacking [87]. While in this setting the potential detrimental effects of transfusions have been confirmed [23,88], it has some peculiarities, including the difficulties in defining ID (see Section 3.2) and the need to minimize delay in elective interventions because of the time required for anemia investigation and treatment. Nevertheless, a recent single-center retrospective study has shown a significant decrease in blood transfusions and an increased 2-year overall survival in cancer patients who underwent surgery after the implementation of a PBM program, as compared to the previous period [89].

Patients with colorectal carcinoma requiring surgery have a particularly high prevalence of ID and ID anemia, and have been more extensively studied. A systematic review of studies on iron supplementation for preoperative anemia in this setting [90] showed a general decrease of blood transfusion rate, but some discrepancies regarding the effect on Hb levels. Indeed, Hb significantly increased in some studies [91], but not in others [92,93], with heterogeneity being possibly attributed to different study design and iron doses. More recently, a multi-center observational study showed that preoperative treatment with FCM significantly reduced transfusion requirements and hospital length of stay [94]. Similar results have been reported by a randomized controlled trial on FCM for the management of preoperative anemia in major abdominal surgery, which mainly enrolled cancer patients [95].

Overall, in line with the proven efficacy of iron for the management of preoperative anemia in elective non-oncologic surgery, these studies suggest that the implementation of IV iron administration protocols may be an effective and safe strategy even in oncologic surgery.

## 6. Possible Risks of Iron Treatment in Cancer Patients: Myth or Reality?

Data from epidemiological studies and animal models have raised some concerns regarding the possible role of deregulated iron metabolism in certain cancer types [96,97], including promotion of tumor growth and enhanced oxidative stress [98]. However, the relevance of such experimental data for cancer patients is limited, since they were typically based on high iron doses, as well as injection routes and iron formulations that are not used in the clinical setting [99].

On the other hand, data from prospective trials evaluating long-term outcomes of IV iron therapy (alone or in combination with ESAs) in anemic cancer patients are relatively scarce. Short-term studies are reassuring, having not revealed an increased tumor progression in patients treated with IV iron and ESAs [54]. In 2015, a prospective randomized controlled trial evaluating treatment with IV iron and ESAs in a small number of patients with hematological malignancies and median follow-up of 1.4 years, did not find any negative effect on long-term outcomes or survival [100]. More recently, a retrospective cohort study, including patients who underwent surgery for colorectal carcinoma with an extended follow-up (median 3.9 years), confirmed that overall and disease-free survivals did not significantly differ in subjects treated with IV iron (FCM in the range of 1000–2000 mg) as compared to a matched group not receiving IV iron [101].

Regarding the risk of infections, no alarming signal has emerged in cancer patients treated with IV iron. Nevertheless, given the role of iron in immune response and microbial proliferation [102], current guidelines prudentially advise to avoid IV iron administration in patients with even suspected active infections [54].

## 7. Available Guidelines: Field of Action, Limitations and Uncovered Issues

**Figure 2:**
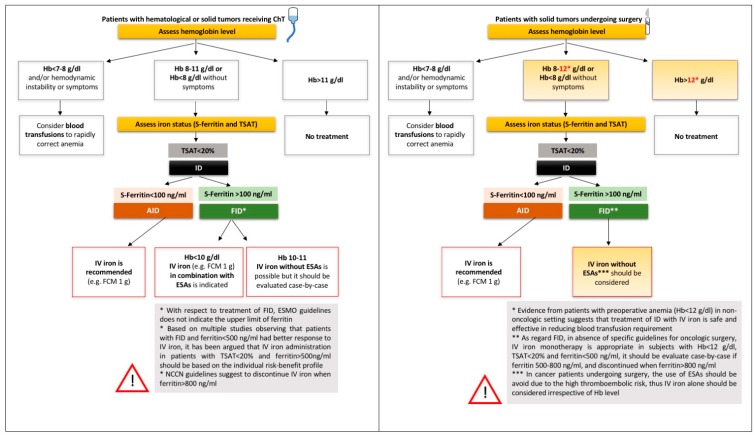
Possible algorithms for the diagnosis and treatment of ID anemia in cancer patients. The proposed algorithms are based on available guidelines for cancer anemia and the most recent clinical evidence regarding preoperative anemia management in the oncologic surgery field. Grey areas underline current uncovered issues.

Early this year, the European Society of Medical Oncology (ESMO) released updated Clinical Practice Guidelines on the management of anemia and iron deficiency in patients with cancer [54]. Accordingly, IV iron is indicated in patients with anemia (Hb <11 g/dL) and AID (defined as serum ferritin <100 ng/mL) or FID (defined as serum ferritin >100 ng/mL, but TSAT <20%) before or during administration of ESAs [54]. The definition of FID is not entirely satisfying, since no upper limit of ferritin is established. It has been argued that in patients with TSAT <20% and ferritin levels >500 ng/mL, the decision regarding iron supplementation should be based on the risk-benefit profile of individual patients, and treatment should be discontinued if ferritin increases above 800 ng/mL [47]. Indeed, the National Comprehensive Cancer Network guidelines suggest that IV iron could be considered for ferritin levels up to 800 ng/mL. However, it has to be noted that such guidelines are largely and explicitly limited to a specific clinical domain, that is patients receiving ChT. To this end, the ESMO guidelines state that “iron therapy should be limited to patients receiving chemotherapy” and discuss some specific conditions like patients receiving cardiotoxic ChT, in whom iron administration should not be synchronous with the anticancer agent [54]. The reason for limiting iron to patients receiving ChT lies on the absence of long-term pharmacovigilance studies in other settings. However, the management of preoperative anemia (see above) represents a notable exception to this prudential rule. Indeed, in this peculiar setting, the primary goal is not to relieve anemia-related clinical symptoms, but rather to reach Hb level able to minimize the risk of blood transfusion. Thus, even a mild preoperative anemia, e.g., Hb 11.5 g/dL in a male cancer patient, is worth being corrected, irrespective of the presence of anemia-related clinical symptoms. Other substantial differences in the perioperative setting are: (1) ESAs should be used with caution, if not avoided, because of the inherent thromboembolic risk temporarily increased by surgery itself; (2) regarding IV iron, it is likely that it would be given just once before the intervention, with limited risk in the long-term follow-up. Notwithstanding the clinical relevance of this peculiar setting (see Section 5), no specific guidelines are available. In particular, no clear cut-off of iron biomarkers has been established to guide iron supplementation. While there is a general agreement on giving IV iron to a cancer patient with even a mild preoperative anemia and AID (again defined as ferritin <100 ng/mL), in cases of FID (defined as TSAT <20%) no consensus exists on the upper ferritin levels at which IV iron should be reasonably administered without risk. This represents a grey area, and there is a clear need of future high-quality prospective trials in the field. Whether or not hepcidin could help in driving iron therapy in cancer patients, as proposed in other settings [63,103], remains to be explored. At our hospital, an ongoing project is evaluating the role of hepcidin in diagnosis and treatment of preoperative anemia in oncologic surgery (Italian Ministry of Health research project no. CO-2016-02361206). Possible algorithms for the diagnosis and treatment of ID in cancer patients, based on the most recent pathophysiological and therapeutic advances in the field, are depicted in Figure 2.

## 8. Concluding Remarks

ID represents a major cause of cancer anemia, especially in patients with gastrointestinal tumors, advanced disease, receiving ChT, and in the perioperative setting. Nevertheless, ID in cancer often remains an overlooked and undertreated condition. This is partly due to difficulties in defining ID based on traditional laboratory biomarkers. Increasing evidence suggests the effectiveness of IV iron administration to treat anemia in cancer patients, alone or in combination with ESAs, in terms of improvement of QoL and reduction of transfusion needs. Further studies on better biomarkers of ID (including hepcidin) and on long-term safety of IV iron administration in this peculiar and challenging condition are required.
